# Inequalities and outcomes: end stage kidney disease in ethnic minorities

**DOI:** 10.1186/s12882-019-1410-2

**Published:** 2019-06-26

**Authors:** Emma Wilkinson, Alison Brettle, Muhammad Waqar, Gurch Randhawa

**Affiliations:** 1 Institute for Health Research, University of Bedfordshire, Luton, Bedfordshire UK; 2 School of Health and Society, University of Salford, Manchester, UK

**Keywords:** Inequalities, Ethnic minorities, Kidney disease, Scoping, Review

## Abstract

**Background:**

The international evidence about outcomes of End Stage Kidney Disease (ESKD) for ethnic minorities was reviewed to identify gaps and make recommendations for researchers and policy makers.

**Methods:**

Nine databases were searched systematically with 112 studies from 14 different countries included and analysed to produce a thematic map of the literature.

**Results:**

Reviews (*n* = 26) highlighted different mortality rates and specific causes between ethnic groups and by stage of kidney disease associated with individual, genetic, social and environmental factors. Primary studies focussing on uptake of treatment modalities (*n* = 19) found ethnic differences in access. Research evaluating intermediate outcomes and quality of care in different treatment phases (*n* = 35) e.g. dialysis adequacy, transplant evaluation and immunosuppression showed ethnic minorities were disadvantaged. This is despite a survival paradox for some ethnic minorities on dialysis seen in studies of longer term outcomes (*n* = 29) e.g. in survival time post-transplant and mortality. There were few studies which focussed on end of life care (*n* = 3) and ethnicity.

Gaps identified were: limited evidence from all stages of the ESKD pathway, particularly end of life care; a lack of system oriented studies with a reliance on national routine datasets which are limited in scope; a dearth of qualitative studies; and a lack studies from many countries with limited cross country comparison and learning.

**Conclusions:**

Differences between ethnic groups occur at various points and in a variety of outcomes throughout the kidney care system. The combination of individual factors and system related variables affect ethnic groups differently indicating a need for culturally intelligent policy informed by research to prevent disadvantage.

## Background

Inequalities in some aspects of End Stage Kidney Disease (ESKD) in the United Kingdom (UK) had been well documented prior to this study. However, to ensure that future research would be based on the most comprehensive evidence this scoping review was commissioned by a national kidney charity in 2017, the topic having been identified through prioritisation process involving people affected by kidney disease as well as clinicians and researchers. This review was the first step in a systematic approach to learn as much as possible from the evidence about inequalities in outcomes for people living and dying with ESKD who are from ethnic minority groups. This paper describes the approach (protocol) used, presents a map of the literature and highlights where there are gaps to be addressed through future research and policy.

Investigating inequalities in kidney care and how they impact upon ethnic minorities is not only necessary for reducing inequalities in the UK but is also likely to benefit the understanding of access to care for other groups with ESKD globally as well. This review therefore took a broad perspective and incorporated the international literature. The objectives of this international systematic scoping review were to:

- identify the evidence base of inequalities in the outcomes of end stage kidney disease for ethnic minorities;
- map the international literature;
- identify gaps and make recommendations for research and policy towards reducing and preventing inequalities in kidney care in the UK and globally.

## Methods

This systematic scoping review was conducted in line with the 5 stage framework outlined by Arksey and O’Malley [1]: protocol and search question development; systematic searching and record management; sifting and refining; charting and mapping. The research question was initially determined via a Delphi study and then refined via discussion within the project team to “Why do ethnic minority patients with End Stage Kidney Failure (ESKD) experience poorer outcomes than non-ethnic minority patients?”.

A systematic scoping review is an early and essential step towards answering the ‘why’ question but necessarily falls short of doing so as it does not apply or build theory for this. It provides a map of the literature, highlights the issues that other researchers have addressed and shows where there are gaps. It follows a systematic protocol as described in this Methods section and offers up observations and recommendations for next steps which are listed in the Conclusion.

The search strategy and literature searches were formulated and refined by EW and AB to be comprehensive and include sources from health, sociology and psychology which considered minority health and any aspect of end stage kidney disease (dialysis, renal replacement therapy, transplant or conservative care and end of life care) in comparison to non-ethnic minorities and in relation to the following outcomes:

- Morbidity, mortality, kidney related, service related, transplant related
- Measures of mental health (e.g. reduction in anxiety or depression using validated tools)
- Measures of emotional well-being or quality of life: (using validated tools).

The definition of ethnic minority was taken from Culley [2] and ethnicity defined as ‘a consciousness of belonging to a particular group based on commonality of family origin and culture of shared values and beliefs which is socially constructed’.

A time frame of 1992 onwards was set to capture evidence from the last 25 years. The searches were wide and sensitive and encompassed a range of thesaurus and free text terms to describe the different descriptors of ethnic minorities and the capture the terms related to end stage kidney failure or disease. Table 1 provides an example of one search strategy for one database.

**Table 1:** Example of one search strategy (Medline database)

1. exp. Kidney Failure, Chronic/ 2. exp. Renal Replacement Therapy/ 3. kidney/ 4. exp. kidney diseases/ 5. exp. renal insufficiency/ 6. exp. renal dialysis/ 7. Glomerular Filtration Rate/ 8. transplantation.mp. [mp = title, abstract, original title, name of substance word, subject heading word, keyword heading word, protocol supplementary concept word, rare disease supplementary concept word, unique identifier, synonyms] 9. 1 or 2 or 3 or 4 or 5 or 6 or 7 or 8 10. Treatment Outcome/ or “Outcome Assessment (Health Care)”/ or outcome assessment.mp. or “Outcome and Process Assessment (Health Care)”/ 11. outcome measure*.mp. [mp = title, abstract, original title, name of substance word, subject heading word, keyword heading word, protocol supplementary concept word, rare disease supplementary concept word, unique identifier, synonyms] 12. exp. Health Status/ 13. exp. “Quality of Life”/ 14. health impact assessment/ 15. well-being.mp. 16. exp. Mental Disorders/ 17. exp. Depression/ 18. Anxiety/ 19. Stress, Psychological/ 20. exp. Dementia/ 21. exp. Cardiovascular Diseases/ 22. end of life care.mp. or Terminal Care/ 23. palliative care.mp. [mp = title, abstract, original title, name of substance word, subject heading word, keyword heading word, protocol supplementary concept word, rare disease supplementary concept word, unique identifier, synonyms] 24. conservative care.mp. [mp = title, abstract, original title, name of substance word, subject heading word, keyword heading word, protocol supplementary concept word, rare disease supplementary concept word, unique identifier, synonyms] 25. mortality.mp. [mp = title, abstract, original title, name of substance word, subject heading word, keyword heading word, protocol supplementary concept word, rare disease supplementary concept word, unique identifier, synonyms] 26. morbidity.mp. [mp = title, abstract, original title, name of substance word, subject heading word, keyword heading word, protocol supplementary concept word, rare disease supplementary concept word, unique identifier, synonyms] 27. survival.mp. [mp = title, abstract, original title, name of substance word, subject heading word, keyword heading word, protocol supplementary concept word, rare disease supplementary concept word, unique identifier, synonyms] 28. 10 or 11 or 12 or 13 or 14 or 15 or 16 or 17 or 18 or 19 or 20 or 21 or 22 or 23 or 24 or 25 or 26 or 27 29. exp. Ethnic Groups/ 30. exp. Continental Population Groups/ 31. exp. Minority Health/ 32. bme.mp. 33. bame.mp. 34. racial.mp. 35. cald.mp. [mp = title, abstract, original title, name of substance word, subject heading word, keyword heading word, protocol supplementary concept word, rare disease supplementary concept word, unique identifier, synonyms] 36. south asian.mp. [mp = title, abstract, original title, name of substance word, subject heading word, keyword heading word, protocol supplementary concept word, rare disease supplementary concept word, unique identifier, synonyms] 37. indo asian.mp. [mp = title, abstract, original title, name of substance word, subject heading word, keyword heading word, protocol supplementary concept word, rare disease supplementary concept word, unique identifier, synonyms] 38. indian.mp. [mp = title, abstract, original title, name of substance word, subject heading word, keyword heading word, protocol supplementary concept word, rare disease supplementary concept word, unique identifier, synonyms] 39. caucasian.mp. [mp = title, abstract, original title, name of substance word, subject heading word, keyword heading word, protocol supplementary concept word, rare disease supplementary concept word, unique identifier, synonyms] 40. exp. African Americans/ 41. exp. Hispanic/ 42. exp. White/ 43. exp. Black/ 44. indigenous.mp. [mp = title, abstract, original title, name of substance word, subject heading word, keyword heading word, protocol supplementary concept word, rare disease supplementary concept word, unique identifier, synonyms] 45. inequality.mp. [mp = title, abstract, original title, name of substance word, subject heading word, keyword heading word, protocol supplementary concept word, rare disease supplementary concept word, unique identifier, synonyms] 46. exp. Health Status Disparities/ or exp. Prejudice/ or exp. Ethnic Groups/ or intersectionality.mp. or exp. “Emigration and Immigration”/ or exp. Social Class/ 47. 29 or 30 or 31 or 32 or 33 or 34 or 35 or 36 or 37 or 38 or 39 or 40 or 41 or 42 or 43 or 44 or 45 or 46 48. exp. research design/ 49. exp. empirical research/ 50. exp. qualitative research/ 51. 48 or 49 or 50 52. 9 and 28 and 47 and 51 53. limit 52 to yr. = “1992 -Current”	

9 electronic databases were searched to identify studies for inclusion in the review (see Table 2 below). There was insufficient resource within the project to search journals and grey literature that could have expanded the potential range of studies to be included.

**Table 2:** Databases searched and number of references retrieved

Database name	Focus	Number of references retrieved
Medline	Biomedical	185
Cinahl	Nursing and allied health	534
Psychinfo	Psychology/Mental Health	78
Web of Science/Web of Knowledge	Science/Social Sciences	224
Scopus	General	577
Social Care Online	Social Care	53
ASSIA	Social Science	370
Global Health	Health	97
Cochrane Database of Promoting Health Effectiveness Reviews	Systematic Reviews	17
Total 9 databases		2135

The searches were undertaken by EW and AB in August 2017 and all records were uploaded on to the Endnote database. Using the inclusion and exclusion criteria shown in Table 3, each record was screened independently on the basis of title by the researcher (EW). A 10% sample of titles were screened by a different member of the research team, any discrepancies resolved, and then the studies were screened by abstract and full text by EW and either included or excluded according to the criteria.

**Table 3:** Inclusion and Exclusion Criteria

Inclusion	Exclusion
● Studies (including systematic reviews and all study designs) that mention ethnic minority health in line with the above definition and in relation to end stage kidney disease and related descriptions (end stage renal failure, dialysis, end of life kidney care). ● Evidence of a measurable outcome on health or wellbeing (e.g. physical or mental health or physiological or quality of life or wellbeing) ● Studies which concern any of the related end stage kidney disease descriptors: ○ Patients on any kind of dialysis ○ Patients on Renal replacement therapy ○ Transplant patients ○ End stage renal failure ● Adults over 18 ● Studies in English, post 1992 ● Any country ● A comparison with non-ethnic minority group (s)	● Studies outside the agreed definition of ethnic minority ● Studies with no measurable outcomes that relate to health ● Studies which do not concern any of the related end stage kidney failure descriptors: ○ Patients on any kind of dialysis ○ Transplant patients ○ End stage renal failure ● Children less than 18 ● Studies in languages other than English, prior to 1992 ● Studies that do not have a comparison with non-ethnic minority group (s) ● Studies that are commentaries, editorials (which do not present data), thought pieces or conference abstracts

**Fig. 1:**
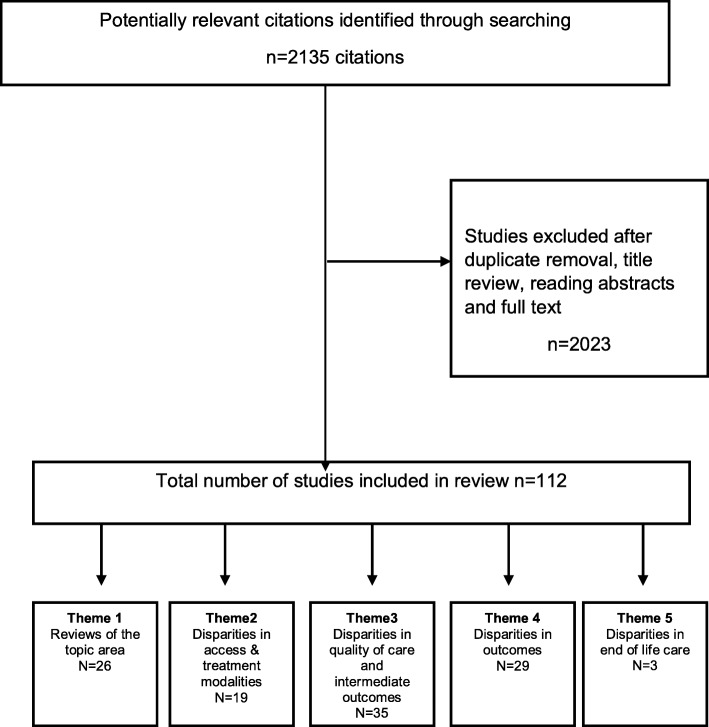
Searching and sifting process

Figure 1 illustrates the searching and sifting process for this review. Data was extracted into a series of evidence tables by EW and MW following project team discussions regarding the column headings and data to be extracted. This included details on author, publication year, author country, ethnic group(s) in focus, research type, research objective, outcomes / indicators and brief summary of findings.

The data was analysed thematically and a map of the data produced to aid analysis, see Fig. 3.

## Results

**Fig. 2:**
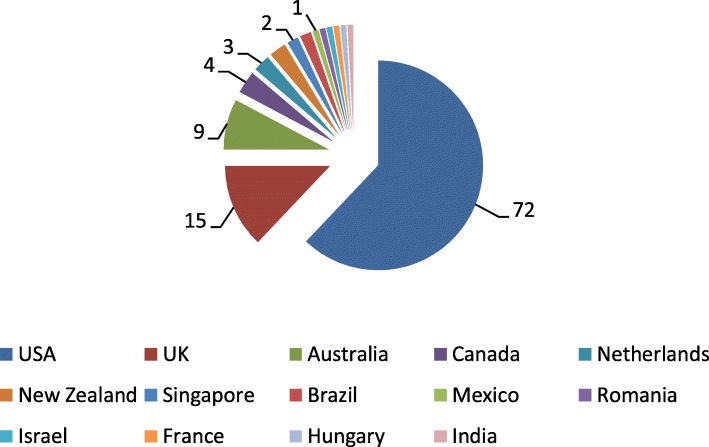
Breakdown of included articles by country of author origin and / or focus

112 studies were included in this review. These focussed on ESKD and included analysis of outcomes for ethnic minorities in 14 different countries listed in Fig. 2 which shows a breakdown of the included papers by author country of origin and / or country focus. The majority of included studies concerned the US (*n* = 72) followed by the UK (*n* = 15), Australia (*n* = 9), Canada (*n* = 4) and the Netherlands and New Zealand (*n* = 3) with the other eight countries (Singapore, Brazil, Mexico, Romania, Israel, France, Hungary and India) being the focus of only one or two studies each.

Included studies related to five different areas or themes which were identified as: an overview of the question topic area in the form of a review (Theme 1); access to a modality of RRT care i.e. access to haemodialysis, peritoneal dialysis, conservative care (Theme 2); care on dialysis and / or being listed for a transplant and moving through the transplant process with a focus on intermediate outcomes (Theme 3); longer term outcomes and survival (Theme 4) and end of life care (Theme 5).

### Theme 1 reviews of the topic area

Twenty-six included studies were reviews, none of which systematic, and they ranged in scope and detail subdividing into five subsets: those with a focus on CKD, hypertensive kidney disease, a general focus, dialysis and transplantation.

#### *Reviews with a focus on CKD* [3–7]

As survival and risk change over time and according to stage and treatment, these studies suggested that interventions should be stratified and tailored in a similar way with prospective longitudinal and cohort research needed which include ethnic minority groups. Furthermore, the multiplicity of factors which put different ethnic minorities at increased risk of CKD and ESKD (hypertension, Type 2 Diabetes Mellitus (T2DM), albuminuria) call for a complex analysis of system-related as well as physiological factors through more cross population multi-levels studies. The urgency of prevention in countries where there are geographical and economic constraints on access to dialysis but increasing incidence in ESKD amongst particular indigenous groups, highlighted the ethical and moral necessities of intervention from a global perspective.

#### *Review with a focus on hypertensive kidney disease* [8]

This paper illustrated the different patterns in ESKD among different ethnic groups within diverse populations which vary from country to country and how ethnicity relates to hypertensive kidney disease as the predominant cause of ESKD for African Americans. The range of mediators which can impact on hypertensive ESKD e.g.: socio-demographics; access to healthcare; blood pressure management; and differences in outcomes on renal replacement therapy (RRT) were described. The earlier onset and more severe disease could be partly explained by worse access to healthcare associated with lower socioeconomic status.

#### *Reviews with a general focus* [9–11]

These studies provide some general observations on inequalities in relation to a variety of different ethnic groups and African and Native Americans. There is more rapid progression from CKD to ESKD in ethnic minorities; minority groups have worse access to care and late referral to specialist renal care. Although transplantation is the ‘best’ treatment for many people, wait time to transplantation is longer for ethnic minorities due to delays in completing the transplant evaluation process and because of lack of suitable matched organs. These three reviews draw attention to the need for culturally competent prevention at the same time as continuing with research to investigate why there are different outcomes seen in ethnic minority groups. The influence of socio-economic factors such as education, social status and associated attitudes towards treatment options for ESKD, and quality of life on RRT compared to majority population groups, are aspects which require better understanding.

#### *Reviews with a focus on dialysis* [12–16]

Five reviews focussed on ESKD from the perspective of dialysis services in the UK, Europe, the US and other parts of the world e.g. Israel. The overarching premise was that the incidence of ESKD and associated need for dialysis is steadily increasing with differences between ethnic groups and associated with changing age structure of populations. These reviews with a focus on different aspects of dialysis care came with the caveat that long term survival of ESKD is very low and survival advantages on dialysis are marginal compared to transplantation with a living donor graft which can extend life considerably but still not back to normal lifespan. Although a survival advantage has been seen in some ethnic groups on dialysis a need for a better understanding of the underlying mechanisms behind the differences and taking action on barriers to access both in the pathway to ESKD but also in access to care on dialysis including transplantation was highlighted.

#### *Reviews with a focus on transplantation* [17–28]

Twelve reviews concerned inequalities in the transplantation process or transplantation outcomes. The overarching premise being that ethnic minorities, although disproportionately represented in patient populations with ESKD, receive fewer transplants and wait longer to receive a transplant. These reviews gave perspectives that varied according to the level of focus: global; country; population; individual as well as stage in the transplant process (pre-emptive transplantation, referral to waiting list, organ allocation, graft survival and immunosuppression) and donation. Overarching conclusions were that better understanding and communication would lead to increased awareness of how the social and individual risk factors interact through the system which is necessary for improving access and reducing inequalities.

### Theme 2 disparities in access & treatment modalities

Nineteen studies reported access to kidney care treatment modalities - dialysis, transplant and conservative care, post diagnosis of ESKD.

Sixteen out of the 19 were cohort designs, 1 observational – cross sectional, 1 mixed method and ethnographic, 1 other (review of registry report data). The studies spanned 6 countries.

Indicators related to access to RRT modalities were: incidence or prevalence on the modality; rate of acceptance; planned and unplanned initiation; time to modality; waiting list probability; time from acceptance onto waiting list to transplantation (and rate); time from start of RRT to transplantation (and rate); transplantation rate.

#### *Access to dialysis – peritoneal dialysis and hemodialysis* [29–37]

Studies described differences in uptake of dialysis modality by different ethnic minority groups compared to majority populations. These were not consistent between different countries and different minority populations highlighting that despite commonalities ethnic minorities are diverse groups whose experiences vary from country to country and on an individual level.

The sociocultural context of renal replacement therapy featured as an important consideration with a focus on possible explanatory factors for the larger prevalent ethnic minority population on dialysis compared to the general population. A third of the countries represented in this review had a national kidney data service which contributed to the research picture by providing quantitative analysis of some elements.

#### *Access to transplantation* [38–47]

Studies showed that ethnic minorities with ESKD are disproportionately represented in the transplantation modality and identified a range of reasons that relate to the different countries’ transplant systems. These included the rates of being listed for transplant; movement from list to transplantation; variations across transplant centres as well in pre-dialysis care and cadaveric and life donation rates, which all showed disparities when ethnic minorities were compared with majority populations. The requirement for future research and service development to take stratified and systemic approaches towards improving access to transplantation to redress these inequalities was raised - as despite being highly technical, dialysis and transplantation are part of a complex process which is influenced by human and sociological factors.

### Theme 3 disparities in quality of care and intermediate outcomes

Thirty-five studies investigated ethnic differences in intermediate outcomes of treatment for ESKD.

Sixteen out of the 35 were cohort designs, 12 other observational (cross sectional, survey, discrete choice experiment); 3 other (programme review, record review, simulation), 3 qualitative and 1 randomised controlled trial (RCT). The studies spanned 6 countries.

Intermediate outcomes related to kidney function were: estimated glomerular filtration rate (eGFR); proteinuria / serum albumin; indicators of quality of care e.g. dialysis dose / dialysis adequacy expressed as Kt/V, urea reduction ratio (URR), anaemia and mineral management, infections, hospitalisation, communication & information; indicators of health and wellbeing or behaviour i.e. nutritional status, quality of life (QOL), coping style; indicators for movement through clinical pathways i.e. through the transplant evaluation and allocation process; transplant indicators e.g. antibody matching, immunosuppression; and indicators of organisational or system function e.g. issues of insurance, allocation priorities, centre level variations, system level intervention.

Ethnic minorities are younger at referral to specialist kidney care and as they often face a longer wait for a transplant so that time spent on dialysis or moving through the transplant process is both an opportunity and a risk for outcomes later in the pathway. The choice of dialysis modality and type of vascular access can be associated with ESKD outcomes and researchers investigated these for disparities amongst ethnic groups.

#### *Quality of care - dialysis* [48–63]

Studies reported on indicators of dialysis care such as hemodialytic dose as well as clinical outcomes such as time to first infection episode and found there these varied by different ethnic groups and required further explanation. Other indicators of maintaining health whilst on dialysis and associated outcomes such as quality of life similarly suggested a stratified approach to identifying related inequalities was necessary as there are age, gender and ethnicity associations to be understood. Links back to wider system variables again were made.

#### *Quality of care– transition to transplant* [64–75]

Ethnic disparities in the transition from diagnosis of ESKD or dialysis to transplant were investigated in 10 of the included studies. The low transplant rates for ethnic minorities which are seen overall in many countries can be linked to disparities further back in the pathway well as availability of suitable donor organs. Barriers responsible for differences in completion of stages and movement through the transplant process were associated with minority ethnicity and linked to levels of information given, knowledge and communication between patient and physician but also within wider networks.

#### *Quality of care – the system* [76–82]

Disparities in the transplant process were associated with ethnicity for some groups and related to interaction with kidney allocation and transplantation systems and influenced by a wide range of factors such as geography, clinician–patient communication, insurance and variation across centres in the same system. These studies highlighted the complexity of transplantation systems.

### Theme 4 disparities in longer term outcomes

Twenty-nine studies focussed on patient survival outcomes for dialysis and transplantation.

Twenty-five out of the 29 were cohort designs and 4 were other observational studies. Collectively they investigated differences in outcomes across 12 broad ethnic groups, 3 analysing by ethnic subgroups. The studies spanned 8 countries.

Longer term outcomes relating to dialysis and transplantation were: survival time on dialysis; time post-transplant; hospitalisation, infection; allograft rejection; quality of life (QOL); and mortality.

The outcomes for dialysis and transplantation varied across different ethnicities, subgroups and stratification by other variables: age, comorbidities (type 1 diabetes mellitus (T1DM), type 2 diabetes mellitus (T2DM), Lupus Nephritis) and location.

#### *Overarching outcomes of ESKD* [83–87]

Studies that reported the overarching outcomes of ESKD identified inequalities faced by particular populations and subgroups drawing attention to the disproportionate burden of dialysis and mortality attributed to rates and risk of diabetes and kidney complications. Other studies highlighted how ethnic inequalities in outcomes can be linked to area differences within countries and across regions.

#### *Dialysis outcomes* [88–101]

Some ethnic minorities have been found to have longer survival on dialysis than the majority population and this apparent survival paradox is against the background of increased risk, faster progression to ESKD and poorer survival in the general population overall. The dialysis survival advantage was not seen to be consistent across the groups studied with different ethnicities and comorbidities; cultural and genetic differences as well as variation in access and delivery of care accounting for this.

#### *Transplantation outcomes* [102–111]

The reduced likelihood of having a transplant is a disparity many ethnic minority patients face and studies investigated risks as well as survival outcomes. Ethnic group and comorbidities were found to be associated with this with greater disparities seen when live and deceased donation were compared. To understand the impact of ethnicity on transplant survival outcomes research investigated other risks underpinning delayed graft function, acute rejection episodes, expanded-criteria donor, impaired graft function which for some ethnicities was an independent risk. Differential rates of risks and survival between ethnic groups were found in some studies.

### Theme 5 disparities in end of life care [112–114]

Three studies that focussed on disparities in end of life care for people with ESKD were included in this review. These studies all came from one country, the US. Two had RCT designs and 1 was a cohort study.

Ethnic minorities were less likely to withdraw from dialysis before death than the majority population with differences explained by sociocultural rather than medical factors. Research in this area included two studies which attempted to understand and improve end of life care for all kidney patients through culturally sensitive intervention.

## Discussion

ESKD is a multifaceted condition with many clinical and sociological elements and there are multiple stages and timings to consider in achieving the best outcome for individuals living with the condition. Within the research literature individuals are generally aggregated into groups to look for evidence of inequalities and this international scoping review has identified that there are disparities between ethnic and non-ethnic minority populations in all aspects of ESKD progression and management.

The search strategy for this review was formulated and conducted by an experienced health researcher (EW) and an expert in literature searching and systematic reviewing (AB). It was designed to be as wide as possible within the resource and time constraints for the work. Tests for publication bias were not conducted nor were reference lists mined or grey literature searched, and these are limitations. However, the research question and search strategy were kept intentionally broad, reviews were included, and the search resulted in studies that used a range of different outcome indicators which related to the different clinical and social elements and stages of care for ESKD. The variety of studies and their juxtaposition within the ESKD field enabled a useful overview of the evidence base, notwithstanding any publication bias.

**Fig. 3:**
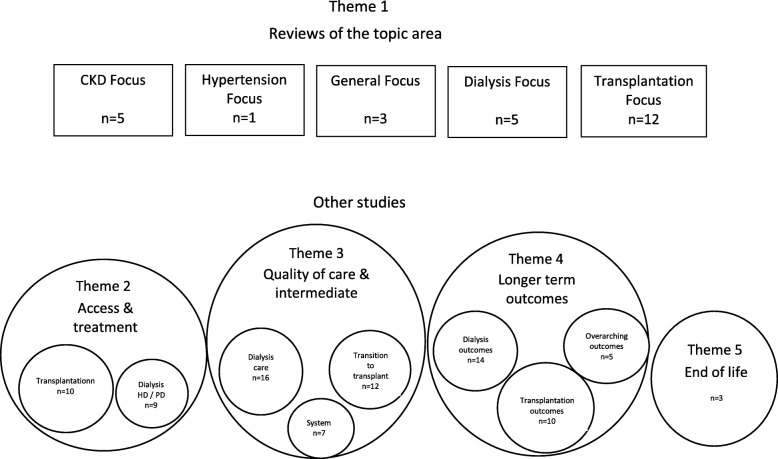
A map of the international literature on comparative studies of minority ethnicity and ESKD

Figure 3 is a visual representation of the main themes in relation to each other and served to highlight some of the gaps. The different themes corresponded to specific elements of the kidney care system – access to treatment modalities, dialysis, transplantation and end of life care – and this review is the first, as far as we are aware, to map the evidence of inequalities in the ESKD field as a whole. This review therefore is a comprehensive starting point for researchers and policy makers to consider where and in what form evidence exists on inequalities in ESKD for ethnic minorities.

Although this review mapped the literature across a schematic ESKD pathway, it highlighted an absence of longitudinal cohort studies (retrospective or prospective) which followed CKD patients as they progress through that pathway. There was also a dearth of studies in two particular areas of ESKD: system-related outcomes and end of life care. The lack of longitudinal and system-related studies may be due to the difficulties of conducting complex systems-related research whereas the lack of end of life studies could reflect a lack of recognition of end of life care as distinct element of kidney care, as well as difficulties in defining end of life and barriers to conducting research in that context.

The literature was also predominantly quantitative and so was limited in helping understand the basis of the differences in outcomes between ethnic groups. Although national registries were a valuable source of data, especially regarding records and analyses of outcomes by ethnic group [13], the national routine datasets do not include indicators for all elements of care, nor analysis at an individual level. Cultural aspects of ethnicity such as attitudes towards death and dying come into play at end of life and there are cultural differences between different parts of healthcare systems so that more qualitative research would help understanding of the individual, cultural and social factors which influence the disparities in outcomes in these and other areas of ESKD. Furthermore, more qualitative and mixed method studies would help to build theories about why inequalities exist and how they can be addressed going forward.

The majority of the evidence for this review has come from a small number of countries and regions notably the US, UK and Australasia. Reasons for the lack of research in other countries, particularly in lower income settings, may reflect how kidney care is funded and delivered in individual countries; the absence of national data sets concerning ESKD; or the lack of ethnicity data. This review highlights the complexities of the kidney and transplant care systems which people with ESKD engage with. Systems in different countries may share some common features but they may also be country specific so that more research and analyses of inequalities in ESKD globally, with cross country comparisons, and a systems approach, would help to share learning and build on this evidence.

Individual countries like the UK, where there is the National Health Service and a national kidney data registry, can not afford to rest on their laurels because as Feehally [9], Lightstone [12] and Randhawa [27] have been suggesting over the past 20 years, the UK’s changing demography towards greater ethnic diversity together with a donation shortage, means that without concerted evidence-based intervention ethnic minorities will continue to experience inequalities in outcomes for ESKD. Furthermore, as ethnic minorities are highly heterogeneous groups, culturally intelligent approaches are needed to understand the barriers and enablers of access within individual country systems and to learn from international comparisons.

## Conclusions – gaps in the evidence

Differences between ethnic groups occur at various points and in a variety of outcomes throughout the ESKD care system. The combination of individual factors and system related variables affect ethnic groups differently indicating a need for culturally intelligent policy informed by research to prevent disadvantage.

This thematic analysis contributes to the international research evidence by mapping the different elements of ESKD research where inequalities for ethnic minorities have been observed. A number of gaps in the international literature have been highlighted which if addressed would strengthen our understanding of why inequalities exist for people from ethnic minority groups who are living and dying with ESKD.

### Gap 1

There is a lack of evidence at all stages in the ESKD pathway and a lack of longitudinal cohort studies (retrospective or prospective) which follow CKD patients as they progress through the pathway.

### Gap 2

The lack of system oriented research into ESKD and a reliance on primary studies using national routine data limits our understanding of how the different elements in the system work together.

### Gap 3

There is a lack of qualitative studies contributing to the evidence base and the literature is predominantly quantitative. This limits a full understanding of the factors that influence disparities in ESKD outcomes.

### Gap 4

There is a lack of evidence from many countries apart from the US, especially low-resource settings, and this limits the possible learning across and between countries.

## Data Availability

Not applicable as this was a review of existing published literature.

## Abbreviations

CKD: Chronic kidney disease
eGFR: Estimated glomerular filtration rate
ESKD: End stage kidney disease
Kt/V: Dialysis adequacy (K = dialyser clearance of urea, t = dialysis time, V = volume of distribution of urea)
QOL: Quality of Life
RCT: Randomised controlled trial
RRT: Renal replacement therapy
T1DM: Type 1 diabetes mellitus
T2DM: Type 2 diabetes mellitus
URR: Urea reduction ratio
